# Human activities aggravate nitrogen-deposition pollution to inland water over China

**DOI:** 10.1093/nsr/nwz073

**Published:** 2019-06-25

**Authors:** Yang Gao, Feng Zhou, Philippe Ciais, Chiyuan Miao, Tao Yang, Yanlong Jia, Xudong Zhou, Butterbach-Bahl Klaus, Tiantian Yang, Guirui Yu

**Affiliations:** 1 Key Laboratory of Ecosystem Network Observation and Modeling, Institute of Geographic Sciences and Natural Resources Research, Chinese Academy of Sciences, Beijing 100101, China; 2 State Key Laboratory of Lake Science and Environment, Nanjing Institute of Geography and Limnology, Chinese Academy of Sciences, Nanjing 210008, China; 3 Sino-French Institute for Earth System Science, College of Urban and Environmental Sciences, Peking University, Beijing 100871, China; 4 Laboratoire des Sciences du Climat et de l’Environnement, CEA CNRS UVSQ, Gif-sur-Yvette 91191, France; 5 State Key Laboratory of Earth Surface Processes and Resource Ecology, Beijing Normal University, Beijing 100875, China; 6 State Key Laboratory of Hydrology-Water Resources and Hydraulic Engineering, Center for Global Change and Water Cycle, Hohai University, Nanjing 210098, China; 7 Institute for Meteorology and Climate Research, Atmospheric Environmental Research, Karlsruhe Institute of Technology, 82467 Garmisch-Partenkirchen, Germany; 8 Department of Civil and Environmental Engineering, University of California, Irvine, CA 92697, USA

**Keywords:** N deposition, N concentration, dam, water area, human activity

## Abstract

In the past three decades, China has built more than 87 000 dams with a storage capacity of ≈6560 km^3^ and the total surface area of inland water has increased by 6672 km^2^. Leaching of N from fertilized soils to rivers is the main source of N pollution in China, but the exposure of a growing inland water area to direct atmospheric N deposition and N leaching caused by N deposition on the terrestrial ecosystem, together with increased N deposition and decreased N flow, also tends to raise N concentrations in most inland waters. The contribution of this previously ignored source of  N deposition to freshwaters is estimated in this study, as well as mitigation strategies. The results show that the annual amounts of N depositions ranged from 4.9 to 16.6 kg · ha^−1^ · yr^−1^ in the 1990s to exceeding 20 kg · ha^−1^ · yr^−1^ in the 2010s over most of regions in China, so the total mass of ΔN (the net contribution of N deposition to the increase in N concentration) for lakes, rivers and reservoirs change from 122.26 Gg N · yr^−1^ in the 1990s to 237.75 Gg N · yr^−1^ in the 2010s. It is suggested that reducing the N deposition from various sources, shortening the water-retention time in dams and decreasing the degree of regulation for rivers are three main measures for preventing a continuous increase in the N-deposition pollution to inland water in China.

## INTRODUCTION

Globally, in the past few decades, atmospheric nitrogen (N) deposition has increased from 16 to 210 Tg N · yr^−1^ [1,2]. This input of N has significantly affected the soil and water chemistry of terrestrial and aquatic ecosystems, increased N_2_O emissions and changed the biological diversity [3–5]. Aquatic ecosystems receive reactive nitrogen (Nr) through leaching from fertilized soils [3,6,7], but also from direct atmospheric deposition on open-water bodies or N transportation caused by N deposition on the terrestrial ecosystem. Although N inputs from non-point-source discharge (including livestock, rural human, urban human and cropland) are dominant, reaching 14.2 Tg N · yr^−1^ wherein agricultural-source discharge with 8.6 Tg N · yr^−1^ accounts for 60.5% [8], direct Nr deposition must also be considered in the Nr budget of freshwaters [3,9,10]. From combustion processes and agricultural activities, Nr deposition in China increased from 13.2 kg · ha^−1^ · yr^−1^ in the 1980s to 21.1 kg · ha^−1^ · yr^−1^ in the 2000s [11], so China has become the largest emitter of Nr to the atmosphere worldwide in the last two decades [10]. The spatial patterns of Nr deposition in the past decades has been obtained from measurements and atmospheric chemistry transport models [12,13], but remains uncertain due to the limited coverage of N-deposition measurement stations and the lack of dry deposition data.

N deposition over inland water should be considered when designing policy options to control N-pollution levels in rivers and lakes. A large number of dams have been built across China in the past three decades, acting to increase the residence time of  N in freshwaters and to decrease streamflow as dam water is used for industry and agriculture [14]. Due to the large number of dam constructions, the effect of dams on the increase in N concentrations in inland waters under N deposition have been gradually augmented, so we address the contribution of changes in the spatial and temporal patterns of N deposition (wet + dry) and the role of the human-controlled stream flow on aquatic N pollution in China.

## RESULTS

### Change of inland-water area and capacity

**Figure 1.**
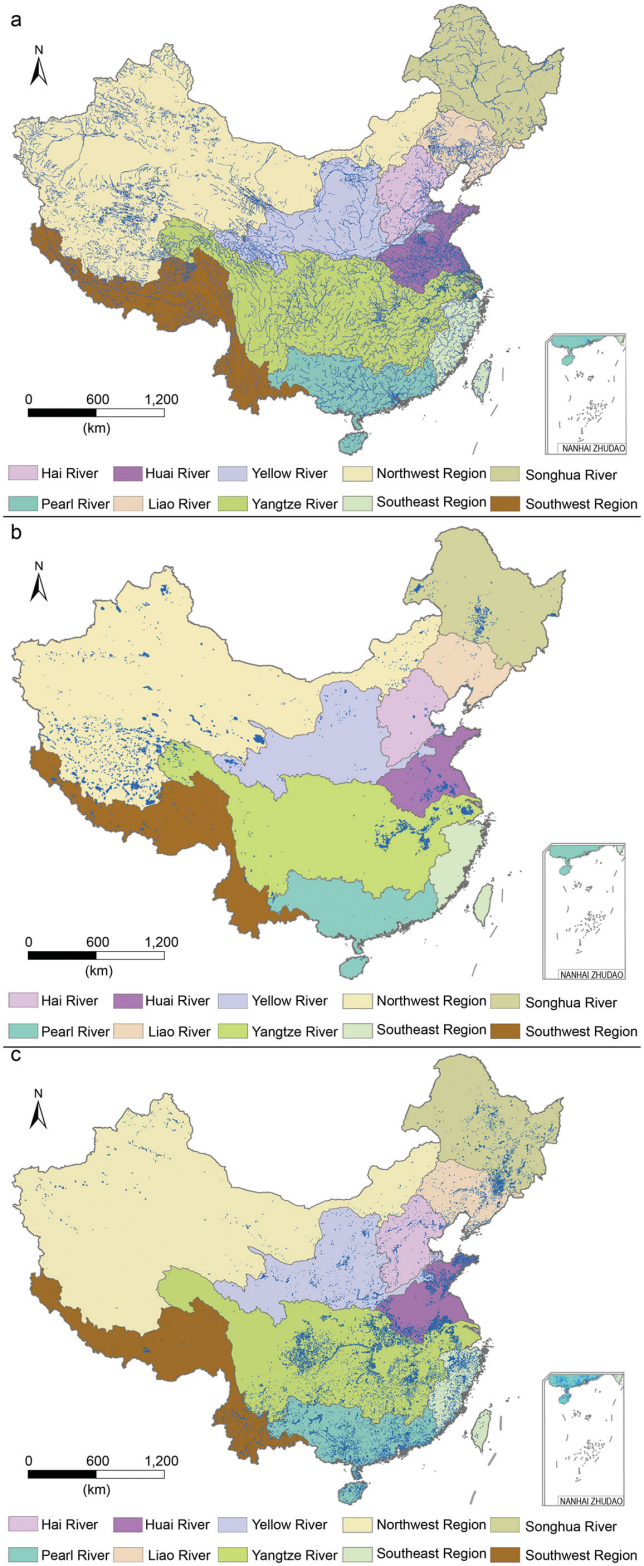
Water-area distribution in different regions in China. (a) Rivers; (b) lakes; (c) reservoirs.

The total surface area of inland water in China increased from 140 444 km^2^ in the 1990s to 146 906 km^2^ in the 2010s, which cover about 1.5% of China's territorial land surface (Fig. 1 and Supplementary Table 1). The area of rivers increased by 108 km^2^, wherein the water areas of the Yangtze River Basin and Huaihe River Basin were 12 973 and 2123 km^2^, respectively, with the highest river density in China (Fig. 1a). More than 50% of natural lakes are located in the Northwest River Basin, especially the Tibet Plateau, covering a total of 39 740 km^2^ of water area. The water area of these lakes has decreased by 318 km^2^ in the past three decades (Fig. 1b), the main reduction areas being in the Yangtze River Basin, Yellow River Basin and Huaihe River Basin, whereas an increase in lake areas occurred in the Northwest basins caused by snow and ice smelt or dam construction [14,15]. Few reservoirs are distributed in Western China, though there are large number of lakes (Fig. 1c). From the 1990s to the 2010s, the reservoir areas have increased by 6672 km^2^, mainly in the Huaihe, Yangtze and Pearl River Basins.

**Figure 2.**
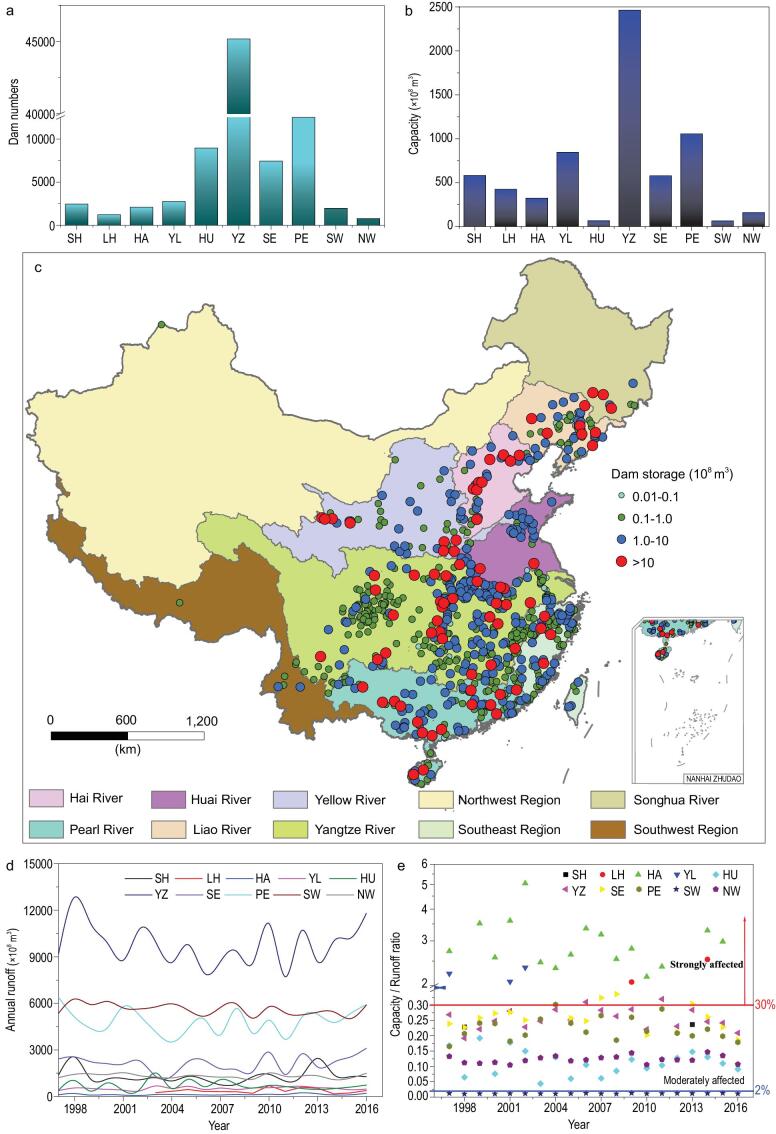
The statistics on dam numbers (a) and storage capacity of dams (b) in different watersheds; the distribution of dams <15 m high in different watersheds in China (c); the annual runoff volume from 1997 to 2016 in different watersheds (d) and the ratio of reservoir capacity to annual runoff discharge from 1997 to 2016 (e), wherein a ratio of reservoir capacity to annual runoff discharge >30% means strongly affected, between 30 and 2% means moderately affected and <2% means not affected [15]. SH, Songhua River Basin; LH, Liaohe River Basin; HA, Haihe River Basin; YL, Yellow River Basin; HU, Huaihe River Basin; YZ, Yangtze River Basin; SE, Southeast River Basin; PE, Zhu River Basin; SW, Southwest River Basin; NW, Northwest River Basin.

Meanwhile, more than 87 000 dams were built, with a total storage capacity of approximately 6560 km^3^ (Fig. 2a and b) in different regions. There are only 773 dams deeper than 15 m, with a total capacity of 4370 km^3^ (67% of total reservoir capacity in China) [16,17] (Supplementary Fig. 1). Most large reservoirs are located in Eastern China, with a clear east-to-west decreased spatial distribution (Fig. 2c). This is because rapid urbanization, high population density and water-resource supply in the Yangtze River Basin, Pearl River Basin and Southeast River Basin accelerated dam constructions (Supplementary Fig. 2), especially dams >15 m in height (Fig. 2c) [16].

In the past two decades, the Yangtze River Basin, Southwest River Basin and Pearl River Basin’ s annual average runoff has always exceeded 300 km^3^, but Haihe River Basin, Liaohe River Basin and Yellow River Basin show a lower annual average runoff, with only 13, 36.7 and 52.1 km^3^, respectively (Fig. 2d) [18]. The ratio of reservoir capacity to annual average runoff defines the degree of regulation (DOR) of a river [19,20]. The Liaohe River Basin (134% of DOR), Haihe River Basin (270% of DOR) and Yellow River Basin (167% of DOR) are showing strongly altered flow regimes, while the Yangtze River Basin (26% of DOR), Pearl River Basin (22% of DOR) and Northwest River Basin (12% of DOR) are moderately affected (Fig. 2e). Although many dams were built in the Yangtze River Basin, Pearl River Basin and Southeast River Basin, they have a lower impact on the annual average runoff.

### N deposition and ΔN over China

**Figure 3.**
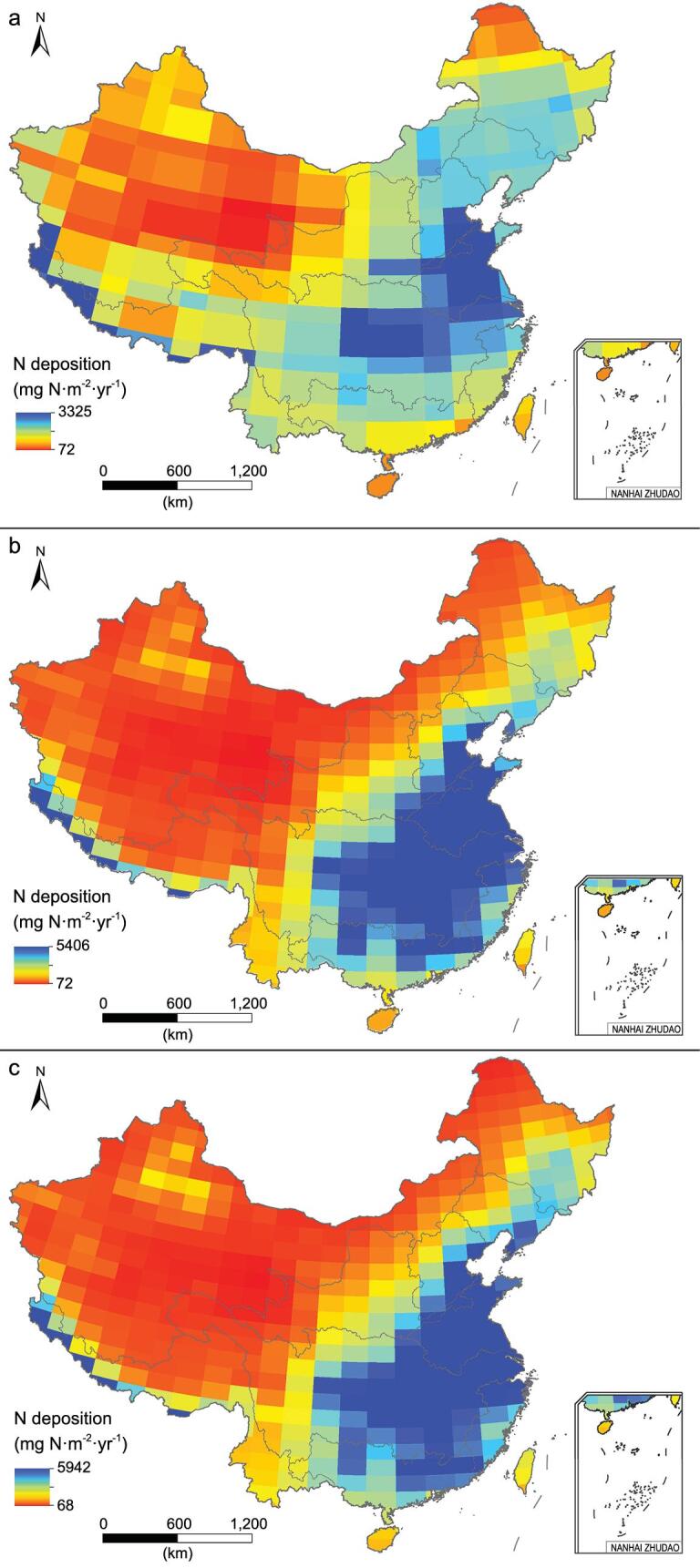
Spatial patterns of N deposition in the 1990s (a), 2000s (b) and 2010s (c) in China (units: mg N · m^−2^ · yr^−1^).

In China, N deposition decreases from southern to western and northern China (Fig. 3). Before the 1990s, the annual amounts of N depositions over China ranged from 4.9 to 16.6 kg · ha^−1^ · yr^−1^ for different regions (Supplementary Table 2) and strongly increased thereafter, especially in the Huaihe River Basin and Yangtze River Basin (Fig. 3a and b). In the 2010s, the annual average N deposition into the Haihe River Basin, Southeast River Basin, Yangtze River Basin and Huaihe River Basin exceeded 20 kg · ha^−1^ · yr^−1^ in rapid-urbanization and high-population-density zones, wherein the Huaihe River Basin and Haihe River Basin N deposition reached 52.9 and 23.4 kg · ha^−1^ · yr^−1^, respectively (Fig. 3c and Supplementary Table 2).

**Figure 4.**
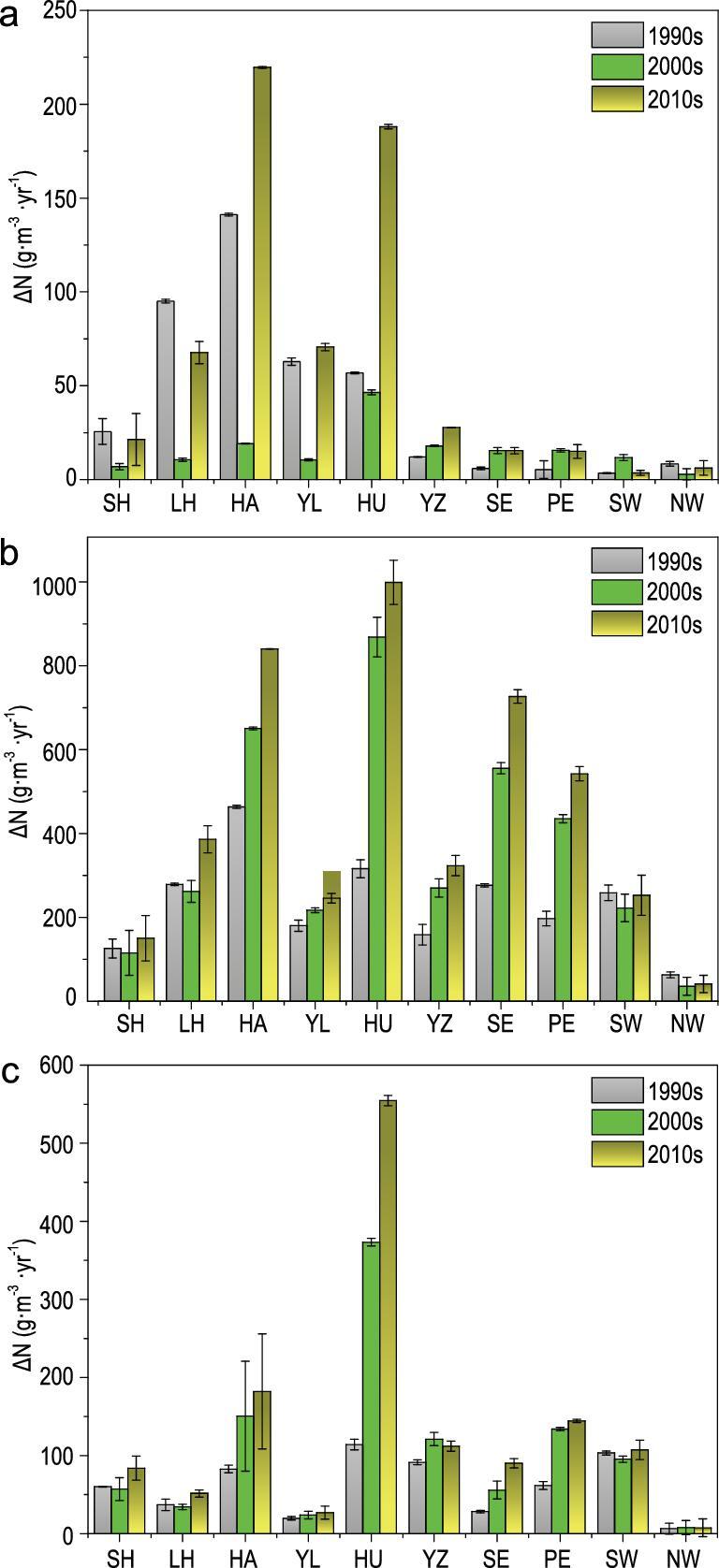
The contribution of N deposition to the increase in N concentration (ΔN) for rivers (a), lakes (b) and reservoirs (c) in different regions. SH, Songhua River Basin; LH, Liaohe River Basin; HA, Haihe River Basin; YL, Yellow River Basin; HU, Huaihe River Basin; YZ, Yangtze River Basin; SE, Southeast River Basin; PE, Zhu River Basin; SW, Southwest River Basin; NW, Northwest River Basin.

In the Haihe River and Huaihe River, we estimated that, between the 1990s and the 2010s, the net contribution of N deposition to the increase in the N concentration (ΔN) of rivers increased by 55 and 229%, respectively. By contrast, the N deposition resulted in small ΔN values in the Yangtze and Pearl River Basins (Fig. 4a). Those differences in ΔN between basins mainly reflect the different residence times of water in rivers, rather than different rates of deposition. From the 1990s to the 2010s, the ΔN of lakes increased mainly in the Huaihe River Basin, Southeast River Basin and Haihe River Basin by 215, 162 and 82%, respectively (Fig. 4b). All these basins are located in industrialized regions with high deposition rates. The large area of lakes collects the input of N deposition, while their small volume, overland flow and subsurface runoff make the residence time of deposited N longer. These processes favor a large increase in N concentrations in lake systems exposed to deposition. All reservoirs exhibit positive ΔN values, the highest being in the Huaihe, Haihe and Yangtze River Basin, being 555 ± 6.5, 182 ± 73.7, and 144 ± 2.2 g · m^−3^ · yr^−1^, respectively (Fig. 4c). The high DOR of reservoirs in the Yangtze River Basin has led to ΔN increased by 23% from the 1990s to the 2010s, but ΔN in the Yangtze River Basin is still very small compared to other basins.

The total mass of ΔN exhibiting a higher change from the 1990s to the 2010s in rivers, increasing by 96%, and in reservoirs, increasing by 181%, than in lakes, which increased by 47% (Supplementary Table 3). The total mass of ΔN for lakes, rivers and reservoirs is far higher in the Yangtze River Basin than in other regions, from 41.0 ± 13.7 Gg N · yr^−1^ in the 1990s to 86.7 ± 15.3 Gg N · yr^−1^ in the 2010s, respectively. Although the higher ΔN occurs in the Huaihe River Basin, Southeast River Basin and Haihe River Basin, the total mass of ΔN for the Southeast River Basin and Haihe River Basin in the 2010s due to the smaller water-surface area only was 0.23 ± 0.17 and 0.23 ± 0.15 Gg N · yr^−1^ for lakes and 5.28 ± 3.82 and 6.23 ± 5.30 Gg N · yr^−1^ for reservoirs, respectively.

## DISCUSSION

### Effect of N deposition on the N cycle in water

Globally, Nr deposition into the terrestrial ecosystem in 2050 will reach 125.2 Tg N · yr^−1^ but Nr transported into rivers, at 149.8 Tg N · yr^−1^, will exceed the Nr-deposition input [1]; moreover, Nr dentification into the terrestrial ecosystem will reach 95 Tg N · yr^−1^, which means that soil and vegetation will be in N-saturation status, so the N-transformation process will accelerate the input into rivers under Nr deposition. In China, Nr deposition into the terrestrial ecosystem was estimated at from 2.7 to 4.6 Tg N · yr^−1^ [21,22], but Nr accumulated in the terrestrial ecosystem and transported to rivers will reach 14.1 and 18.1 Tg N · yr^−1^, respectively, which also indicates that Nr deposition accelerates N transformation and transport. Globally, ∼25% of 230 Tg N · yr^−1^ of Nr inputs into the terrestrial area, wherein 11 Tg N · yr^−1^ were transported into inland waters or drylands, not transported into coastal areas, and 48 Tg N · yr^−1^ were transported in rivers to coastal systems [1].

As N deposition increases, the N-retention and -enrichment capacity for the ecosystem would reach its threshold and then be in N-saturated status, so any additional N deposition would lead to N being lost to streams, groundwater and, eventually, the atmosphere [3,23]. N leaching caused by N deposition is restricted not only by the N-saturation limit, but also by topography and geomorphology. The N-saturation limit mainly depends on microbial and plant demands [13,24], wherein soil properties and plant uptake are important parameters. About 5–67% of the N deposited into the terrestrial ecosystem is transported into inland water by leaching [25,26], which exhibits huge range and regional differences in N retention. In the present study, we estimate that the N wet and dry deposition into the terrestrial ecosystem ranges from 4.3 to 18.3 Tg N · yr^−1^, but the total mass of ΔN for lakes, rivers and reservoirs has only ranged from 122.3 Gg N · yr^−1^ in the 1990s to 237.8 Gg N · yr^−1^ in the 2010s (Supplementary Table 3).

**Figure 5.**
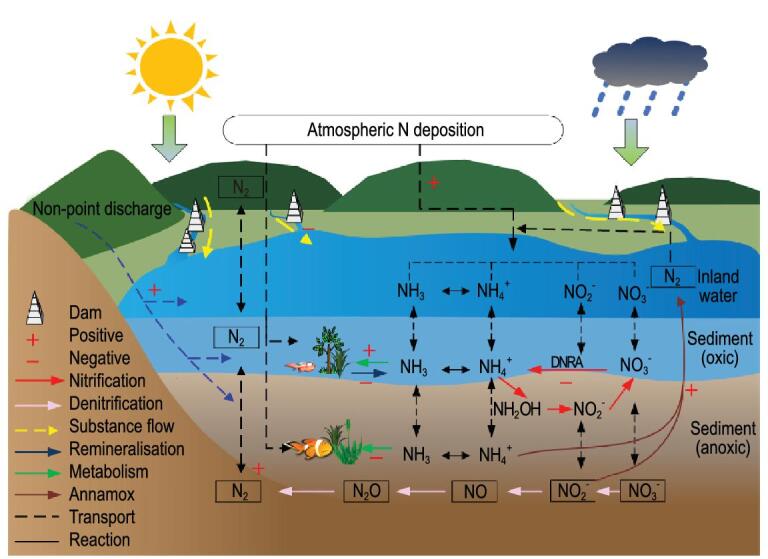
Mechanism on the effect of N-deposition increase and dam construction on the N cycle in inland water. (Note: As the yellow arrow shows, before constructing dams, the waterbody can quickly flow and the runoff path length and water surface are changing as the water level rises and falls, so the retention of N deposition and associated material and energy will be limited. On the contrary, after constructing dams, the water only internally flows in the reservoirs formed by dams, so water can not naturally flow downstream. When the reservoir discharges flood or is fully stored, the N can freely flow downstream. In addition, the water-surface area obviously enlarges and gradually becomes stable, so most N deposition will be detained and accelerate the internal N cycle.)

The denitrification and anammox processes are two main pathways for N removal during N transportation from streams into rivers, lakes and reservoirs, which affects the N balance in inland waters (Fig. 5). Han *et al*. reported that denitrification in the Yangtze River comprised 20–32% of the N-removal rates [27], whereas streams or rivers in the USA showed higher N-removal rates, at ∼30–70% [28,29]. Globally, an average of 18% of N enters waters that can be denitrified [30]. Overall, denitrification in waters primarily depends on temperature, residence time and depth of water. The anammox process in waters is also mainly regulated by the temperature, residence time and depth of the water [31,32], organic carbon levels [33,34], substrate availability [35,36] and salinity [37]. Zhu *et al*. reported that an average of 20% of N_2_ production was related to an anammox reaction in the Three Gorge Reservoir in China [38], whereas the anammox rater in a typical inland water for Lake Tanganyika was only 13% of N_2_ production [39]. Although the N denitrification and anammox processes for inland water show different zonal change due to environmental change, an average of 50–90% of ΔN would be removed directly by those two processes in inland water over China [27–29,38]. The rest of the ΔN would go into the internal N cycle by metabolism and sediment, so only a small part of the ΔN impacts on increasing the N concentration in water.

In the present study, the water area increased by dam construction enhanced ΔN, while the increase in water depth shortened the transport path for N not-point discharge (Fig. 5). Moreover, as an increase in N deposition and its flow is detained, rivers, lakes and reservoirs provide a continuum with substantial capacity for denitrification under anoxic environments, including nitrate, abundant organic matter, and sediments and suspended particulate [40,41]. However, under dam construction, ΔN enhanced by the increase in N from non-point discharge, sediment and atmospheric N deposition combined with decreasing detrend of N mineralization and nitrification would be far higher than ΔN decreased by denitrification and anammox reaction. In addition, the decrease in dissimilatory NO_3_–N reduction to NH_4_–N (DNRA) mainly depended on organic-matter availability, so high organic-matter loading would mitigate the importance of the denitrification process and stimulate nitrate reduction to ammonium (Fig. 5) [42,43].

### Human-activity impact on the N cycle

Before the 1980s, the N concentrations in inland waters were lower than 1.0 mg N · L^−1^ but, after the 1990s, most waters increased rapidly and exceeded 15 mg N · L^−1^ [8,16], mainly from increased anthropogenic sources, such as industrial waste, croplands, human and livestock excrement, and N deposition [8,21]. In addition, nitrate concentration in groundwater mainly from agricultural and domestic sources also increased sharply after the 1980s [44], being close to the class IV standard in 2015 [45]. Although P was proved to be a limited nutrient on phytoplankton and algal [46], the ratio of TN/TP was used to co-determine the limiting nutrient for water eutrophication [47,48]. Therefore, expediting the N flow and cycle period in waters would be an effective pathway to decrease N concentrations, but dam construction would impact on the water temperature and salinity [49], enhance the storage of N by increasing the residence time of water and delay the N release to downstream rivers, resulting in increased concentrations of  N and the nutrients cycle, not only in water, but also in sediments [20].

The DOR can be used as an indicator to estimate the increased residence time of  N in dams. Water resources regulated by reservoirs under anthropogenic activities are far higher in China than the global average of 7.6% of the world's rivers [16], except the Southwest River Basin (Fig. 2e). More than 90% of Chinese rivers show a water-resource excessive-exploitation risk with the higher 2% of the DOR threshold [20]. The average water residence time in the heavily dammed Yangtze River Basin is 0.28 years [15], while the average water-cycle period of natural flow for rivers is only 0.033 years [50,51]. A practical way will be to shorten the water-retention time in dams—that is, decrease the DOR. Though it maybe decreases the hydroelectricity service for the local economic benefit [49], it would be more helpful for N transport and transfer in the water environment. Although enhancing the reservoir-storage capacity to increase water availability would be conflict with the decrease in the DOR, if local water resources are plentiful, we can properly enhance the DOR; otherwise, we should decrease the DOR and improve water quality by wastewater treatment, reducing pollutants and water reuse [52].

As ‘Hu Huanyong’ line phenomena showing, the highly developed and densely populated region can be distinguished by a geographic boundary in China (Supplementary Fig. 2) [53,54]. Most human activities, such as industrial or agricultural emissions or discharge, hydroelectricity by dams, agricultural irrigation and aquaculture, are more concentrated on the east ‘Hu Huanyong’ line, which could lead to more lakes or rivers disappearing or shrinking, land reclamation and lake isolation. The main purpose of dams is to supply irrigation water for agriculture, aquaculture and other water usage. In order to stop the continuous increase in the N-deposition pollution to inland water due to human activities, China now needs more flexible regional water strategies and water-management solutions. Though human activity for agricultural irrigation, aquaculture and hydroelectricity services by regulating the DOR can gain economic benefit in the short term, in the long term, sacrificing the water-environment quality to promote local economic development, especially in the hydroelectricity service, would make us pay more to remediate water pollution in the future. Therefore, enhancing the water-cycle rate for alleviating nutrient eutrophication and keeping biodiversity and a free-flow path will be more important in maintaining a healthy aquatic ecosystem.

## METHODS

### Data sources of the dams and water resources

All data on the reservoir spatial characteristics including the location, reservoir capacity and build year were retrieved from the Global Reservoir and Dam (GRanD v1.1; http://www.gwsp.org/products/grand-database.html) database [16]. The GRanD compiles the reservoirs with a storage capacity of more than 0.1 km³ and contains 6862 records globally and 773 reservoirs in China. The statistical data of the distribution and reservoir capacities of dams are summarized by the Chinese water conservancy yearbook [17].

The water-resources statistics for different regions were retrieved from the China Water Resources Bulletin published by the Ministry of Water Resources of the People's Republic of China from 1997 to 2016 [18]. The water-resources data for years before 1997 were computed using a linear-regression approach. The linear relation was built between the measured discharge at significant hydrological gauges and the water-resources statistics after 1997 for each basin. All the measured discharge data were collected from the Hydrological Bureau.

### Data sources for inland-water-area changes in China

The data related to the water-surface changes during the 1990s to the 2010s were retrieved from the Annual Report on Sensing Monitoring of Global Ecosystem and Environment (large terrestrial water area) issued by National Remote Sensing Center of China. The land-use data of China in the 1990s, 2000s and 2010s were from the land-use database developed by the Chinese Academy of Sciences. This database is a multi-temporal land-use dataset with a mapping scale of 1:100 000, which was interpreted from Landsat TM/ETM images by manual visual interpretation [55–59].

### N-deposition fluxes over China

Both the dry and wet deposition fluxes of all N species were quantified by the global aerosol chemistry climate model LMDZ-OR-INCA, which couples online the LMDz (Laboratoire de Météorologie Dynamique, version 4) General Circulation Model [60] and the INCA (IN teraction with Chemistry and Aerosols, version 4), an aerosol module [61]. To run the model, emissions data included oceanic emissions for N (NH_3_), vegetation emissions for N (NO), agricultural activities (including fertilizer use and livestock) for N and fuel combustion for both N (NO_y_ and NH_x_). Regarding N-containing aerosols and gases, LMDZ-INCA was run with a fully interactive atmospheric N cycle [61] at a horizontal resolution of 1.27° latitude by 2.5° longitude with 39 vertical layers in the atmosphere to simulate the dry and wet deposition of NO_y_ and NH_x_ for 1980, 1990 and 1997–2013. Meteorological fields from a reanalysis of the European Centre for Medium-Range Weather Forecasts (ECMWF) have been used in the present configuration to nudge the model transport and removal processes for 1980 and 1990 and for each year during the recent 1997–2013 period. Before using these observational data for model evaluation, we further collected data to increase the coverage of wet N-deposition data from the National Acid Deposition Monitoring Network (NADMN) established by the China Meteorological Administration and a full description of model performance for a 1° × 1° grid of N-deposition flux can be found in [13].

To evaluate the modeled N-deposition rates, we used a recent global data set of wet N-deposition rates measured during 2002–06 [62]. Dry N-deposition data were from previous estimation [63,64]. Before using these observational data for model evaluation, we further collected data to increase the coverage of wet N-deposition data in South America and Africa. A full description of model performance can be found in [13]. The modeled spatial distributions of N wet deposition were evaluated by *in situ* measurements globally. The comparison shows that the spatial patterns in the observed deposition data can be captured by our model. The coefficient of correlation (*R*^2^) of the log-transferred deposition rates is 0.55 for N wet deposition, indicating a significant correlation between models and data (*P* < 0.001) (Supplementary Fig. 3).

### N concentration increased from deposition

In order to estimate the net contribution of N deposition to the increase in N concentration (ΔN) without calculating N-transformation and -transport processes after N entering into the inland water, we first calculate the N deposition on waterbodies. The annual total N deposition on rivers, lakes and reservoirs are calculated as:


(1) \begin{equation*}{{\rm{N}}_{{\rm{tyr}}}} = {{\rm{N}}_{{\rm{dep}}}} \times {\rm{A}}\end{equation*}

where N_dep_ is the unit area of the N deposition on rivers, lakes and reservoirs; A is the area of rivers, lakes and reservoirs in different regions; and N_tyr_ is the total mass of the N_yr_ deposition on rivers, lakes and reservoirs in different regions [65].

In this study, we calculate the ΔN by the ratio of the amounts of N_dep_ directly entering into waterbodies to the capacity for rivers, lakes and reservoirs, so the N-deposition impact on N concentrations in inland water is calculated as:


(2) \begin{equation*}\Delta {\rm{N}} = {{\rm{N}}_{{\rm{tyr}}}}/{{\rm{W}}_{\rm{C}}}\end{equation*}

where ΔN is the N concentration in water enhanced by the N deposition; and W_C_ is the total capacity of rivers, lakes and reservoirs in different regions.

The total capacity of rivers and reservoirs were extracted from the Chinese water conservancy yearbook [17] and the Ministry of Water Resources of the People's Republic of China [18]. The total capacity of lakes was calculated by empirical equations according to Yang and Liu's method [15]. Lake storage is primarily based on the surface area; the cumulative storage of the selected lakes reached 72% of the total estimated lake-storage volume, so the selected lakes can be a reasonable representation of all of China's lakes. The equations established for lakes were calculated using Equation (1):


(3) \begin{equation*}{\rm{Lakes}}:{\rm{C}} = 1.2601{{\rm{A}}^{1.1726}}( {{{\rm{R}}^2}{\rm{ = }}0.7711})\end{equation*}

where C is the lake-storage volume in 10^6^ m^3^ and A is the surface area in km^2^.

### Uncertainty analysis

There are three possible contributors to the uncertainty in estimating the water area including rivers, lakes and reservoirs. First, some uncertainty is from the data sources of land-use change in China. Although there may be some errors from the classification and boundary determination of different land-use types in interpreting remote-sensing images, they have more than 95% of the qualitative accuracy rate and were widely used in China [56]. Second, errors derive from the water-area statistics in 10 hydroclimatic zones in China. Some rivers, lakes and reservoirs are across different zones, so this may generate some errors in calculating the water area of each zone. However, this uncertainty has little effect on the objective of this work due to the very few water bodies that exist in the boundary of different zones. Furthermore, the interannual variation and seasonal dynamics of water bodies may also contain uncertainties. For example, the changing rate of expanded and shrunken lakes were about 6.2 to 18.7 and –1.6 to 16.3%, respectively, in the lakes of Tibetan Plateau from 2009 to 2014, of which the coefficients of variation in different seasons of lake area were about 0.17∼0.43% due to the change in rainfall and temperature [66].

In order to obtain long-term N depositions, the estimation is subject to several sources of uncertainty. First, the constant N species for each emission source from measurements need to consider the spatial variability of N species across China. Second, the emission of dust from land-cover change and its impact should be more considered in the future, when regional sources are better understood [67,68]. Third, the daily atmospheric input from 1980 to 2013 due to heavy computational load would lead to the monthly resolution of nutrient deposition being unable to capture episodic transport [69].

The normalized mean bias (NMB) is of –8% for N wet deposition. A statistical analysis showed that 50% of data are subject to a bias of –25 to 50% in the modeled wet N deposition. The observed wet N-deposition data enable us to further evaluate the modeled wet deposition of N in the oxidized form (NO_3_) and reduced (NH_4_) forms by region (Supplementary Fig. 4). The NMB of modeled wet NO_3_ deposition is –12% in North America, –31% in Europe and –28% in East Asia. According to a previous study [70], the NMB of wet NO_3_ deposition is –28, 13 and –54% over the three regions, respectively. In addition, the NMB of our modeled wet NH_4_ deposition is 20% in North America, –30% in Europe and –28% in East Asia, compared with –32, –4.5 and –60%, respectively, in the previous study [70]. The negative bias in the N deposition over East Asia is likely due to underestimation in the emissions of reactive N in this region from the ACCMIP inventory [71,72], which was used in that study. Here, we updated the emissions related to reactive N with the latest inventories from the ECLIPSE GAINS.4a inventory [73] and confirmed this hypothesis.

## Supplementary Material

nwz073_Supplemental_File
