# Supplementary material for: Human activities aggravate nitrogen-deposition pollution to inland water over China
Source: Natl Sci Rev. 2019 Jun 25;7(2):430–40. doi: 10.1093/nsr/nwz073 (PMC8288964; doi:10.1093/nsr/nwz073)
Supplement: nwz073_Supplemental_File [file nwz073_supplemental_file.docx]

 **Fig.S1 Dams number (a) and storage capacity (b)statistics with deeper than 15m in China**

**Fig.S2 The population (a), water resource (b) and dam distribution with more 15 m height (c), wherein the “Hu Huanyong” line is defined as a geographic boundary between the high-developed and densely-populated Eastern region and the less-developed and sparsely-populated Western region in China by the name of a famous geographer in China in 1935.**

The population distribution was extracted from Fu et al’s (Population Grid_China) (<http://www.geodoi.ac.cn/doi.aspx>? doi=10.3974/geodb.2014.01.06.v1.).

**Fig.S3 The coefficient of correlation (R^2^) of log-transferred deposition rates**

**Figure S3. Comparison of modeled and observed wet deposition of different forms of N in North America (a), Europe (b), Asia (c), Africa (d), South America (e) and other regions (f). Data measuring the total deposition of reduced and oxidized forms of N are shown as grey crosses. The numbers in bracket show the normalized mean bias of log-transferred deposition rates with the numbers of data in bracket.**

**Table S1. Surface area change of rivers, lakes, and reservoirs between the 1990s and 2010s in China (unit: km^2^)**

| Zone | 1990s | | | 2000s | | | 2010s | | |
| --- | --- | --- | --- | --- | --- | --- | --- | --- | --- |
|  | Rivers | Lakes | Reservoirs | Rivers | Lakes | Reservoirs | Rivers | Lakes | Reservoirs |
| Haihe | 1516.61 | 82.10 | 2180.31 | 1496.86 | 138.98 | 2680.71 | 1482.87 | 99.44 | 2659.09 |
| Liaohe | 1876.60 | 787.58 | 1623.12 | 1894.23 | 860.14 | 1689.95 | 1846.44 | 808.92 | 1816.58 |
| Northwest | 2326.32 | 38599.34 | 1373.86 | 2520.14 | 39457.63 | 1656.68 | 2527.56 | 39740.17 | 1375.36 |
| Songhua | 3612.32 | 9012.58 | 1913.03 | 3510.49 | 8102.45 | 2025.91 | 3480.12 | 7703.42 | 2299.78 |
| Southeast | 1853.20 | 100.20 | 1962.67 | 1812.19 | 98.77 | 2113.19 | 1784.55 | 112.86 | 2549.07 |
| Southwest | 1470.03 | 3884.52 | 147.12 | 1481.91 | 3909.72 | 162.67 | 1483.11 | 3844.22 | 161.62 |
| Yangtze | 12630.28 | 15360.29 | 10818.33 | 12747.78 | 15269.43 | 11161.01 | 12973.38 | 15114.11 | 12282.43 |
| Yellow | 3479.83 | 2499.05 | 1567.70 | 3021.45 | 2492.07 | 1715.22 | 3289.13 | 2468.23 | 2206.64 |
| Pearl | 3959.47 | 424.14 | 6140.21 | 3886.44 | 423.85 | 7007.57 | 3863.79 | 426.17 | 7193.17 |
| Huaihe | 2021.71 | 3963.00 | 3259.05 | 2062.43 | 4344.39 | 3915.46 | 2123.49 | 4076.73 | 5114.11 |

**Table S2. Trends in Nr deposition in different regions between the 1990s and the 2010s**

**(Unit: kg N.ha^-1^. yr^-1^)**

| Zone | 1990s | | | 2000s | | | 2010s | | |
| --- | --- | --- | --- | --- | --- | --- | --- | --- | --- |
|  | Mean | Min | Max | Mean | Min | Max | Mean | Min | Max |
| Haihe | 12.50 | 10.72 | 16.80 | 19.21 | 7.26 | 37.16 | 23.42 | 8.54 | 43.53 |
| Liaohe | 11.11 | 10.46 | 11.65 | 10.59 | 5.55 | 16.1 | 15.46 | 6.86 | 25.88 |
| Northwest | 4.91 | 0.88 | 29.05 | 2.80 | 0.72 | 18.48 | 3.25 | 0.68 | 21.8 |
| Songhua | 7.65 | 3.10 | 11.42 | 6.87 | 2.41 | 15.77 | 8.89 | 15.3 | 22.32 |
| Southeast | 7.72 | 4.32 | 10.49 | 15.47 | 7.22 | 20.62 | 20.70 | 10.22 | 25.21 |
| Southwest | 13.57 | 4.36 | 33.25 | 11.69 | 2.29 | 30.33 | 13.24 | 2.5 | 34.21 |
| Yangtze | 10.56 | 2.71 | 24.68 | 17.95 | 1.36 | 36.35 | 21.46 | 1.45 | 45.18 |
| Yellow | 8.77 | 1.63 | 16.8 | 10.56 | 1.09 | 32.92 | 11.91 | 1.16 | 39.36 |
| Pearl | 7.07 | 3.28 | 12.23 | 15.59 | 5.46 | 24.42 | 19.44 | 7.4 | 26.93 |
| Huaihe | 16.64 | 13.95 | 17.64 | 46.47 | 40.61 | 54.06 | 52.85 | 47.46 | 59.42 |

**Table S3. Nr input via deposition to rivers, lakes and reservoirs in different regions between the 1990s and the 2010s**

**(Unit:** **Gg N. yr^-1^)**

| Zone | 1990s | | | 2000s | | | 2010s | | |
| --- | --- | --- | --- | --- | --- | --- | --- | --- | --- |
|  | Rivers | Lakes | Reservoirs | Rivers | Lakes | Reservoirs | Rivers | Lakes | Reservoirs |
| Haihe | 1.90±0.92 | 0.10±0.05 | 2.73±1.32 | 0.21±0.18 | 0.27±0.22 | 5.15±5.02 | 3.47±2.19 | 0.23±0.15 | 6.23±5.3 |
| Liaohe | 2.08±0.22 | 0.87±0.09 | 1.80±0.19 | 0.23±0.20 | 0.91±0.91 | 1.79±1.78 | 2.85±2.51 | 1.25±1.04 | 2.81±2.45 |
| Northwest | 1.14±1.06 | 18.93±11.2 | 0.67±0.59 | 0.36±0.27 | 11.06±10.38 | 0.46±0.42 | 0.82±0.68 | 12.91±2.08 | 0.45±0.44 |
| Songhua | 2.76±2.13 | 6.89±7.29 | 1.46±1.19 | 0.74±0.54 | 5.57±2.78 | 1.39± 1.20 | 3.09±2.77 | 6.85±5.19 | 2.04±2.03 |
| Southeast | 1.43±1.14 | 0.08±0.06 | 1.52±1.21 | 2.91±2.43 | 0.15±0.13 | 3.27±2.83 | 3.69±2.68 | 0.23±0.17 | 5.28±3.82 |
| Southwest | 1.99±1.26 | 5.27±4.98 | 0.20±0.13 | 6.62±2.45 | 4.57±4.45 | 0.19±0.17 | 1.96±1.03 | 5.09±4.85 | 0.21±0.13 |
| Yangtze | 13.34±7.75 | 16.22±3.75 | 11.42±3.77 | 16.60±14.60 | 27.41±13.42 | 20.04±19.04 | 27.85±16.73 | 32.44±16.09 | 26.36±13.71 |
| Yellow | 3.05±2.75 | 2.19±2.13 | 1.37±1.09 | 0.54±0.45 | 2.63±1.20 | 1.81±1.65 | 3.92±2.03 | 2.94±2.03 | 2.63±2.07 |
| Pearl | 2.80±1.54 | 0.30±0.28 | 4.34±3.54 | 7.19±7.37 | 0.66±0.50 | 10.92±3.29 | 7.51±6.55 | 0.83±0.73 | 13.99±12.05 |
| Huaihe | 3.36±0.74 | 6.60±1.46 | 5.42±1.20 | 3.66±2.33 | 20.19±4.91 | 18.19±4.43 | 11.22±2.31 | 21.55±4.43 | 27.03±5.55 |
| Sum | 33.86±19.51 | 57.46±31.29 | 30.94±14.23 | 39.09±30.82 | 73.42±38.9 | 63.22±39.83 | 66.40±39.48 | 84.32±36.76 | 87.02±47.55 |
